# Effects of Periodontal Therapy on Blood Lipid Levels in 10 Dogs With Periodontitis and Hyperlipidemia

**DOI:** 10.1155/crve/9088922

**Published:** 2025-06-16

**Authors:** Yu Sahashi, Miwako Sahashi, Yoshiaki Hikasa

**Affiliations:** ^1^Sahashi Veterinary Hospital, Inagawa, Hyogo, Japan; ^2^Joint Department of Veterinary Medicine, Faculty of Agriculture, Tottori University, Tottori, Japan

**Keywords:** dog, hyperlipidemia, lipoproteins, periodontal therapy, periodontitis

## Abstract

This study is aimed at evaluating the effects of periodontal therapy on blood lipid levels in 10 dogs with both periodontitis and hyperlipidemia. A retrospective analysis was conducted on data from 10 client-owned dogs with mild to severe periodontitis and hyperlipidemia. Dental procedures such as periodontal probing and intraoral radiography, followed by scaling, curettage, and polishing, were performed. The teeth were extracted, and the gingival mucosa was sutured in five cases of severe periodontitis. Hematological and general biochemical tests and lipoprotein (i.e., chylomicrons, very low-density lipoprotein (VLDL), low-density lipoprotein, and high-density lipoprotein) analyses were performed before and 4 weeks after periodontal treatments. Consequently, periodontal treatments significantly reduced the total triglyceride, VLDL-triglyceride, and total cholesterol levels. This study suggested that periodontitis treatment effectively ameliorates hyperlipidemia in dogs with both periodontitis and hyperlipidemia.

## 1. Introduction

Periodontal disease is commonly encountered in daily canine practice [1]. It occurs in approximately 90% of all canine patients [1]. Its systemic ramifications have been extensively reported in human literature. Evidence in the veterinary literature on the negative effects of periodontal disease on systemic health is increasing [2] because of periodontal tissue inflammation caused by the bacteria in the dental plaque at the interface between the teeth and gingival tissues [3]. Human medicine has recognized the association between periodontal and systemic diseases. Periodontal disease has been reported to adversely affect systemic health and exacerbated by chronic systemic inflammation [4]. It adversely affects patients with diabetes [5, 6], obesity [7, 8], aspiration pneumonia [9], preterm low birth weight [10], transient ischemic attack and stroke [11], rheumatoid arthritis [12, 13], and renal failure [14]. Oral hygiene was found to be associated with cardiovascular disease severity, tumors, Type 2 diabetes, and respiratory disease in humans [15].

In veterinary medicine, research has emphasized the relationship between periodontal and systemic diseases [16]. For example, *Porphyromonas gulae fimA genotype C*, a suspected canine periodontal disease pathogen, was associated with mitral regurgitation [17, 18]. Chronic azotemic kidney disease was also associated with periodontal disease severity in dogs [19]. Periodontal treatment effectively controls glycemic levels in dogs with diabetes mellitus [20]. A retrospective cohort study of factors associated with lifespan in pet dogs reported that the mortality risk decreased with increasing dental scaling frequency [21], demonstrating an association between periodontal and systemic diseases in dogs.

Hyperlipidemia commonly occurs in dogs, and its prevalence may increase with aging-related diseases. It can be primary and secondary to other diseases [22]. Primary hyperlipidemia is less common and is usually associated with certain breeds, including hypertriglyceridemia in Miniature Schnauzers and hypercholesterolemia in Shetland Sheepdogs [22]. Secondary hyperlipidemia is more common and is associated with several underlying etiologic conditions such as endocrine diseases including hypothyroidism, diabetes mellitus, and hyperadrenocorticism, cholestasis, protein-losing nephropathy, obesity, and high-fat diet [22]. In addition to underlying diseases, hyperlipidemia can result in complications such as pancreatitis, hepatobiliary disease, atherosclerosis, ocular disease, and seizures in dogs [22]. Therefore, hyperlipidemia control may be important to alleviate secondary systemic diseases.

A retrospective cohort study in humans demonstrated that severe periodontal disease is a risk factor for the onset of cardiovascular diseases such as acute myocardial infarction and hypercholesterolemia-related stroke [23]. In humans with both periodontal disease and hyperlipidemia, periodontal treatment was reported to improve the serum lipoprotein levels [24, 25]. However, no studies have reported the effects of periodontal treatment on hyperlipidemia in dogs. Therefore, this study is aimed at evaluating the influence of periodontal therapy on blood lipid levels in dogs with both periodontal disease and hyperlipidemia.

## 2. Case Presentation

### 2.1. Cases

Ten client-owned dogs with both periodontal disease and hyperlipidemia (hypercholesterolemia and/or hypertriglyceridemia) that were treated at the Sahashi Veterinary Hospital were analyzed in this study. Ethical approval from a committee was not required. Informed consent was obtained from the dog owners. Their signs, complications, and medication history are summarized in Table 1. Periodontal disease severity in pets was evaluated based on the established stages [26]. Three, two, and five dogs were classified as Stages II, III, and IV (severe), respectively, according to the periodontal disease scoring system reported by Harvey et al. [27]. Hyperlipidemia was defined as plasma or serum total triglyceride levels of ≥ 100 mg/dL and/or total cholesterol levels of ≥ 200 mg/dL after at least 8 h of fasting. In addition to chronic periodontal disease and hyperlipidemia, complications such as obesity, dermatitis, bladder stones, gallbladder mucocele, renal failure, mitral insufficiency, and epilepsy occurred in 6 of 10 dogs (Table 1). Four dogs were treated with some medications (Table 1). They continued the intake of previous medications and commercially available foods without changes before and after periodontal treatment. Four dogs (Cases 6, 7, 8, and 10) received daily care, including toothbrushing, but the other dogs did not. Cases 1, 6, and 10 underwent routine dental procedures for maintenance. In contrast, halitosis occurred in Cases 2, 3, 4, 5, 7, 8, and 9, and Cases 2, 7, and 8 additionally resisted having their mouths touched. Cases 7 and 9 presented with sneezing and purulent nasal discharge, while Case 2 had drooling and eating difficulty.

### 2.2. Periodontal Disease Treatment

Each dog was administered a mixture of 0.2 mg/kg of butorphanol (Meiji Seika Pharma Co., Ltd., Tokyo, Japan) and 0.2 mg/kg of midazolam (Fuji Pharma Co., Ltd., Tokyo, Japan) intravenously for sedation and analgesia. A 2-mg/kg of alfaxalone (Meiji Seika Pharma) was intravenously administered to induce anesthesia, and the dogs were intubated with a cuffed endotracheal tube. Anesthesia was maintained with isoflurane (MSD Animal Health, Tokyo, Japan) in oxygen through a semiclosed circle system under spontaneous ventilation. Periodontal probing and intraoral radiography, followed by scaling, curettage, and polishing, were the dental procedures performed. Teeth were extracted in accordance with the extraction criteria for periodontal disease in dogs [1], and five cases with severe periodontitis underwent gingival mucosa sutures (Cases 2, 5, 7, 8, and 9). The surgeon was an experienced veterinary dentist. Bupivacaine (Sandoz Pharma, Basel, Switzerland) was administered as a local anesthetic to perform the tooth extraction. After periodontal treatment, clindamycin (Virbac Japan, Tokyo) of 5 mg/kg (p.o., q12 h) was administered for 4–7 days in all dogs to prevent infection.

In the periodontal treatment, five cases did not undergo tooth extraction (Cases 1, 3, 4, 6, and 10). In the Triadan system, 205 and 206 in Case 2 and 210 and 105 in Case 5 were extracted. In Case 7, a total of 12 remaining teeth (Triadan 102, 103, 104, 108, 204, 208, 304, 309, 402, 403, 404, and 409) were extracted. After canine and molar extractions, a gingival mucosa flap was created and sutured with a 4–0 synthetic absorbable material (Ethicon, Inc, United States). In Case 8, a total of 13 teeth (Triadan 105, 110, 205, 206, 207, 210, 301, 302, 310, 401, 402, 403, and 410) were extracted. In Case 9, all four remaining teeth (Triadan 104, 204, 304, and 404) were extracted. The incisors were extracted using the closed technique [28], whereas the canines and molars were extracted using the open technique [28]. After extraction, a gingival flap was created and sutured with a 4–0 synthetic absorbable material. No wound healing problems occurred postprocedure.

### 2.3. Blood Sampling and Analyses

Venous blood samples (5 mL) were collected from the cephalic vein immediately before (pre) and 4 weeks after postperiodontal treatment, after fasting for at least 8 h each. An automated hematology analyzer (pocH-100iV, Sysmex, Hyogo, Japan) was used to measure complete blood counts. Plasma and serum were separated and used for general biochemical tests and lipoprotein analyses, respectively. General biochemical tests were carried out using a dry chemistry analyzer (Fuji Drychem 4000 V, Fuji Film Medical, Tokyo, Japan) for the plasma samples. Lipoprotein analyses were performed using a gel-filtration high-performance liquid chromatography system (LipoSEARCH; Immuno-Biological Laboratories Co, Ltd, Japan) for the serum samples. The cholesterol and triglyceride levels were measured in four major classes of lipoproteins, that is, chylomicrons, very low-density lipoprotein (VLDL), low-density lipoprotein (LDL), and high-density lipoprotein (HDL).

### 2.4. Data Analyses

For simplicity, pre and postperiodontal treatment data are expressed as means ± standard deviations in tables and were analyzed for normality using the Shapiro–Wilk test and statistical significance with the paired *t*-test in normally distributed data or with the Wilcoxon matched-pair signed-rank test in nonnormally distributed data. The significance level for each analysis was defined as *p* < 0.05.

### 2.5. Results in Hematological and Biochemical Variables

Approximately 40% of the dogs that underwent dental treatment were diagnosed with hypercholesterolemia and/or hypertriglyceridemia. Ten of them were followed up.  Table 2 presents changes in hematological and biochemical variables. White blood cell counts and C-reactive protein (CRP) levels were not significantly different between before and after periodontal treatment. Changes in total cholesterol and total serum triglyceride levels after the periodontal treatment varied among cases. The dry chemistry analysis demonstrated significantly (*p* = 0.043) lower total cholesterol levels at posttreatment than at pretreatment. The LipoSEARCH analysis revealed lower VLDL cholesterol levels (*p* = 0.093) posttreatment than pretreatment; however, no significant change was observed. The dry chemistry analysis revealed lower plasma total triglyceride levels at posttreatment than at pretreatment; however, the difference was not significant (*p* = 0.089). The LipoSEARCH analysis demonstrated significantly lower total triglyceride levels (*p* = 0.003) at posttreatment than at pretreatment. Regarding the lipoprotein fractions, the VLDL-triglyceride level was significantly lower (*p* = 0.009) posttreatment than pretreatment. HDL triglyceride levels also showed a decreasing trend at posttreatment than at pretreatment; however, the difference was not significant (*p* = 0.073). Other plasma biochemical variables, namely, aspartate aminotransferase, alkaline phosphatase, glucose, blood urea nitrogen, and creatinine, did not show significant differences between pre and posttreatment.

The results in eight dogs, excluding two Miniature Schnauzers, which are known to be predisposed to hyperlipidemia, are also shown in Table 3. The dry chemistry analysis revealed significantly lower total cholesterol and triglyceride levels (*p* = 0.047 and 0.008, respectively) posttreatment than pretreatment. The LipoSEARCH analysis demonstrated that total triglyceride and VLDL-triglyceride levels were significantly (*p* = 0.02 and 0.02, respectively) lower posttreatment than pretreatment. These results excluding Miniature Schnauzers were similar to the results in 10 dogs, including this breed.

## 3. Discussion

This study suggested that periodontal treatment, including tooth extraction and scaling, may reduce the total triglyceride and VLDL-triglyceride levels. In addition, the total cholesterol levels by the dry chemistry analysis significantly decreased after periodontal treatment. This study noted some discrepancies in total cholesterol and total triglyceride levels between the dry chemistry and LipoSEARCH analyses. In lipid measurements, the inclusion of free glycerol (FG) can cause variabilities in the values [29]. In the LipoSEARCH analysis, as FG is fractionated separately and not included in the lipid measurement fraction, the lipid value is expected to be lower than in the analytical method including FG; however, the lipid concentration can be measured more accurately. Conversely, because the enzymatic measurement method in the dry chemistry analysis includes FG, the lipid concentration may be slightly higher [29]. In the present study, no significant differences in total triglyceride and total cholesterol levels were noted between the dry chemistry and LipoSEARCH analyses, although the mean total cholesterol level with the dry chemistry analysis was slightly (12–17 mg/dL) higher than that with the LipoSEARCH analysis. On the other hand, total cholesterol and total triglyceride levels are generally expected to not be different between serum and plasma samples. In this study, as the plasma and serum samples were analyzed with different methods using dry chemistry and LipoSEARCH, respectively, their differences in total cholesterol and triglyceride levels cannot be simply compared. However, the present study found no significant difference between the serum and plasma samples in total cholesterol or total triglyceride levels at pre and posttreatment.

Several studies have reported an association between periodontitis and hyperlipidemia in rodents and humans [24, 25, 30, 31]. A previous study in mice demonstrated that periodontal infection with *Porphyromonas gingivalis*, a representative periodontopathic bacterium, is associated with high LDL cholesterol levels [30]. A human study also reported that periodontal therapy decreased the serum LDL cholesterol, total cholesterol, and triglyceride concentrations with a reduced local inflammation and tissue destruction [25]. In patients with periodontitis and hyperlipidemia, intensive periodontal treatment reduced the serum triglyceride and proinflammatory cytokine levels, that is, tumor necrosis factor-alpha (TNF-*α*), interleukin (IL)-1*β* (IL-1*β*), and IL-6 [24]. In the present study, periodontitis treatments, including tooth scaling and extractions, improved periodontal health and reduced inflammation, possibly with the help of the use of antibiotics, which might have contributed to the improvement of hyperlipidemia in dogs. However, no significant changes in the leukocyte counts and CRP levels were observed probably due to the chronic nature of periodontitis in the present cases because CRP is more elevated in acute inflammation. However, a previous study reported that increased CRP levels and white blood cell counts occurred during active periods of periodontal disease [32]. Another study reported that the periodontal disease severity affected the CRP levels and renal function and that CRP levels decreased after periodontal treatment [33]. In our study, as some dogs with low periodontal disease severity were included, no significant difference in CRP levels may have been observed. In addition, some dogs included in this study had bladder stones or atopic dermatitis, which may have led to less reduction in inflammation. Therefore, investigating the relationship between inflammation and hyperlipidemia using other inflammatory markers, including high-sensitivity CRP, TNF-*α*, and IL-1*β*, is necessary.

The inclusion of hyperlipidemic-prone breed and obese dogs in this study may have been partly responsible for the elevated blood lipid levels in dogs with periodontal disease. However, periodontal disease appears to be a contributing factor to the worsening of hyperlipidemia even in these dogs because periodontal disease treatment reduces hyperlipidemia. In addition, in this study, two dogs were administered pravastatin and fenofibrate, which directly affect blood lipid levels, and one dog was administered ursodeoxycholic acid, which may indirectly affect blood lipid levels, while the other drugs did not affect lipid levels. However, as the dosage of these drugs was not changed during the observation period, their effects on the blood lipid levels before and after periodontal treatment were likely to be minimal. To address the small sample size and the associated higher risk of Type I error, adjustments were made to the statistical analysis. The Shapiro–Wilk test was employed to assess normality, and appropriate statistical methods were used based on the results. When variance differences were detected, variance-specific methods were used to ensure robustness and reduce the likelihood of Type I errors. This approach accounted for discrepancies in the variance and distribution, maintaining the validity of the analysis. However, further studies, including larger sample sizes and stricter significance levels, may be needed to reduce the risk of Type I errors.

The exact mechanism by which periodontal disease exacerbates hyperlipidemia remains unknown. However, anaerobic bacterial infections occur in periodontal disease. Anaerobic bacterial infections induce local and systemic increases of proinflammatory cytokines including TNF-*α*, IL-1*β*, and IL-6 [34]. These inflammatory cytokines can cause lipid metabolism changes, including increased LDL cholesterol and triglyceride levels, through increased hepatic lipogenesis, adipose tissue lipolysis, or decreased blood clearance [35]. In addition, previous studies in humans have reported that periodontal therapy, including scaling and antibiotic administration, decreased the proinflammatory cytokines such as TNF-*α*, IL-1*β*, and IL-6 [36, 37]. Therefore, the attenuation of canine hyperlipidemia by periodontal therapy in this study may be responsible for the inflammatory cytokine levels associated with periodontal treatment.

In conclusion, this study suggested that periodontal treatment may ameliorate hyperlipidemia in dogs. However, the results of this study may be cautiously interpreted due to the small and heterogeneous sample size. Further studies on the changes in blood inflammatory cytokine levels may be needed to elucidate the relationship between chronic periodontitis and lipid metabolism in dogs.

## Figures and Tables

**Table 1:** Signs, complications, and medication history of 10 dogs with both hyperlipidemia and periodontal disease.

**Case**	**Breed**	**Age (years)**	**Sex**	**Body weight (kg)**	**Stages of periodontal disease ** ^**‡**^	**Triglyceride (mg/dL)**	**Total cholesterol (mg/dL)**	**Other complications**	**History of medications**
1	Toy Poodle	6	Male^a^	6.2	II	236	315	Obesity	None
2	Jack Russell Terrier	15	Female^†^	5.5	IV	121	450	Renal failure (IRIS Stage 2)	None
3	Pomeranian	9	Male^a^	4.8	III	247	328	Gallbladder mucocele, bladder stones	Ursodeoxycholic acid (9.6 mg/kg, p.o., q24 h) for > 1 year; fenofibrate (2.8 mg/kg, p.o., q24 h) for > 1 year
4	Toy Poodle	10	Female^†^	4.0	III	311	371	None	None
5	Toy Poodle	11	Female^†^	4.3	IV	500	297	Atopic dermatitis	None
6	Border Collie	6	Female^†^	14.8	II	41	347	None	None
7	Miniature Schnauzer	12	Female^†^	7.2	IV	238	148	Mitral insufficiency	Alacepril (1.7 mg/kg, p.o., q24 h) for 2 years
8	Miniature Schnauzer	11	Female^†^	7.0	IV	362	157	Idiopathic epilepsy	Zonisamide (3.5 mg/kg, p.o., q12 h) for > 1 year
9	Toy Poodle	9	Female^†^	4.3	IV	250	167	None	None
10	Miniature Dachshund	10	Male intact	6.5	II	22	450	None	Pravastatin (0.76 mg/kg, p.o., q12 h) for > 5 years

Abbreviation: IRIS, International Renal Interest Society.

^a^Castrated.

^†^Ovariohysterectomized.

^‡^Severity stages are based on the classification reported by Bauer et al. [23].

**Table 2:** Hematological and blood biochemical values before and after periodontal treatment in 10 dogs.

**Variables**	**Before periodontal treatment**	**4 weeks after the periodontal treatment**	**p** ** value** ^**a**^
White blood cells (× 10^3^/mm^3^)	9.98 ± 2.85	10.33 ± 3.05	0.46
C-reactive protein (mg/dL)	0.66 ± 0.12	0.59 ± 0.37	0.53
Total cholesterol (mg/dL)^†^	303 ± 113	280 ± 91	0.043
Total cholesterol (mg/dL)^‡^	286 ± 120	268 ± 90	0.25
CM cholesterol (mg/dL)^‡^	2.4 ± 3.0	1.3 ± 1.5	0.38
VLDL cholesterol (mg/dL)^‡^	20.3 ± 17.8	16.3 ± 14.1	0.093
LDL cholesterol (mg/dL)^‡^	69.1 ± 56.3	56.6 ± 40.0	0.21
HDL cholesterol (mg/dL)^‡^	194 ± 78	194 ± 64	0.96
Triglyceride (mg/dL)^†^	233 ± 145	169 ± 146	0.089
Triglyceride (mg/dL)^‡^	278 ± 195	165 ± 125	0.003
CM triglyceride (mg/dL)^‡^	31.3 ± 39.4	11.2 ± 14.4	0.16
VLDL-triglyceride (mg/dL)^‡^	215 ± 157	134 ± 111	0.009
LDL triglyceride (mg/dL)^‡^	22.7 ± 15.4	14.4 ± 7.3	0.10
HDL triglyceride (mg/dL)^‡^	9.1 ± 5.7	5.8 ± 3.4	0.073

Abbreviations: CM, chylomicrons; HDL, high-density lipoprotein; LDL, low-density lipoprotein; VLDL, very-low-density lipoprotein.

^a^Data between the two groups were analyzed using either the paired *t*-test or the Wilcoxon matched-pair signed-rank test after the Shapiro–Wilk normality test.

^†^Measurements were performed by dry chemistry analysis.

^‡^Measurements were performed by LipoSEARCH analysis.

**Table 3:** Hematological and blood biochemical values before and after periodontal treatment in eight dogs (excluding Miniature Schnauzers).

**Variables**	**Before periodontal treatment**	**4 weeks after the periodontal treatment**	**p** ** value** ^**a**^
White blood cells (× 10^3^/mm^3^)	10.06 ± 3.03	10.58 ± 3.10	0.51
C-reactive protein (mg/dL)	0.66 ± 0.10	0.64 ± 0.40	0.93
Total cholesterol (mg/dL)^†^	341 ± 90	312 ± 69	0.047
Total cholesterol (mg/dL)^‡^	320 ± 108	297 ± 72	0.26
CM cholesterol (mg/dL)^‡^	1.1 ± 1.5	1.0 ± 1.3	0.87
VLDL cholesterol (mg/dL)^‡^	17.5 ± 19.0	12.4 ± 13.0	0.15
LDL cholesterol (mg/dL)^‡^	82.8 ± 54.8	68.8 ± 54.8	0.28
HDL cholesterol (mg/dL)^‡^	219 ± 65	216 ± 51	0.94
Triglyceride (mg/dL)^†^	216 ± 156	120 ± 93	0.008
Triglyceride (mg/dL)^‡^	227 ± 185	127 ± 105	0.02
CM triglyceride (mg/dL)^‡^	13.8 ± 15.6	8.3 ± 11.2	0.55
VLDL-triglyceride (mg/dL)^‡^	186 ± 164	100 ± 95	0.02
LDL triglyceride (mg/dL)^‡^	19.2 ± 15.4	13.7 ± 7.6	0.38
HDL triglyceride (mg/dL)^‡^	7.1 ± 4.6	5.1 ± 3.4	0.31

Abbreviations: CM, chylomicrons; HDL, high-density lipoprotein; LDL, low-density lipoprotein; VLDL, very-low-density lipoprotein.

^a^Data between the two groups were analyzed using either the paired *t*-test or the Wilcoxon matched-pair signed-rank test after the Shapiro–Wilk normality test.

^†^Measurements were performed by dry chemistry analysis.

^‡^Measurements were performed by LipoSEARCH analysis.

## Data Availability

The data that support the findings of this study are available on request from the corresponding author. The data are not publicly available due to privacy or ethical restrictions.
